# Cancer cell reprogramming: turning the enemy into an ally

**DOI:** 10.1038/s41392-024-02102-w

**Published:** 2025-01-17

**Authors:** Severin Guetter, Kaiji Fan, Hendrik Poeck

**Affiliations:** 1 Department of Internal Medicine III, Hematology & Internal Oncology, University Hospital Regensburg, Regensburg, Germany; 2 Leibniz Institute for Immunotherapy (LIT), Regensburg, Germany; 3 Bavarian Cancer Research Center (BZKF), Regensburg, Germany

**Keywords:** Immunotherapy, Cancer therapy, Antigen processing and presentation

In a recent study published in *Science*, Ascic and colleagues^1^ present a novel approach to reprogram tumor cells in vivo into immunogenic, conventional dendritic cell (cDC)-like cells, directly triggering an immune response within the tumor microenvironment (TME). Their findings provide a promising novel strategy conquering the challenges of ex vivo cell modification.

For many years, the approach to cancer treatment has shifted from solely destroying cancer cells with chemotherapy or radiation to focusing on activating the patient’s immune system or providing it with the tools needed to effectively eliminate cancer on its own. The success of these immunotherapies relies on the priming of tumor specific T cells within the TME or the tumor draining lymph nodes (tdLN). This is achieved by presentation of tumor antigens by specialized antigen-presenting cells (APCs) on major histocompatibility complex (MHC) molecules to the T cells. APCs not only activate T cells but also recruit them to the site of action being the TME or tdLN where the T cells get in contact with their enemies and kill them by the release of cytotoxic effector molecules like interferon-γ (IFN-γ) and Granzyme B.^2^

In recent years there has been increasing evidence that type I cDCs (cDC1) are required to prime tumor-reactive T cells within the TME, especially in patients treated with immune checkpoint inhibitors.^3^ cDC1s are a rare type of dendritic cells that, once mature, display high levels of MHC-I, MHC-II, CD40, and lineage-specific markers XCR1 and CLEC9A. In tumors, they are essential for attracting and activating T cells by secreting chemokines and cross-presenting antigens, which helps to drive an immune response against cancer. Despite these key functions, cDC1s have not yet been widely utilized in immunotherapy. However, this concept was already addressed in a publication of Salmon and colleagues, where they enhanced the tumor immunogenicity by the targeted manipulation of cDC1 precursors in the tumor.^4^ Providing cDC1s to cancer patients from alternative sources, rather than relying on their own precursors, could help overcome the limitations of immune checkpoint blockade. This strategy may address challenges such as insufficient T cell priming in an immune-suppressed TME, where APCs and their precursors are lacking.

To address this need, the authors developed an adenoviral platform to deliver the transcription factors PU.1, IRF8, and BATF3, collectively termed PIB, directly into tumor cells. These factors could reprogram tumor cells in vivo into cDC1-like cells with antigen-presenting functions (Fig. 1). By this approach they were able to transform cancer cells into a source of immune activation directly within the TME. To assess immune responses and tumor regression after reprogramming, murine cancer models (YUMM1.7, LLC, and B16) and human cancer models (e.g., glioblastoma, melanoma, and breast cancer spheroids, as well as xenografts) were used. In vivo, PIB adenoviruses transformed tumor cells into cDC1-like cells, which expressed immunogenic markers (MHC I/II, CLEC9A, XCR1) and attracted T cells to the TME. This “reprogramming” induced tertiary lymphoid structures containing T and B cells, boosting polyclonal cytotoxic T cell infiltration and promoting tumor regression. Systemic immunity was also triggered, eliminating tumor cells injected on the opposite flank. In the B16 melanoma model, reprogramming even provided long-term immunity, with melanoma-specific CD8^+^ central and effector memory cells detected months later. Reprogramming of human glioblastoma spheroids also successfully generated CD45^+^ HLA-DR^+^ cDC1s, despite the presence of immunosuppressive cells like cancer-associated fibroblasts, myeloid-derived suppressor cells (MDSCs), or pericytes. However, this approach may not fully overcome tumor-driven immune evasion mechanisms, such as the production of prostaglandin E2 or transforming growth factor-β, recruitment of MDSCs, secretion of interleukin-10, and the increase of regulatory T cells and tumor-associated macrophages, all of which warrant further investigation.

**Fig. 1:**
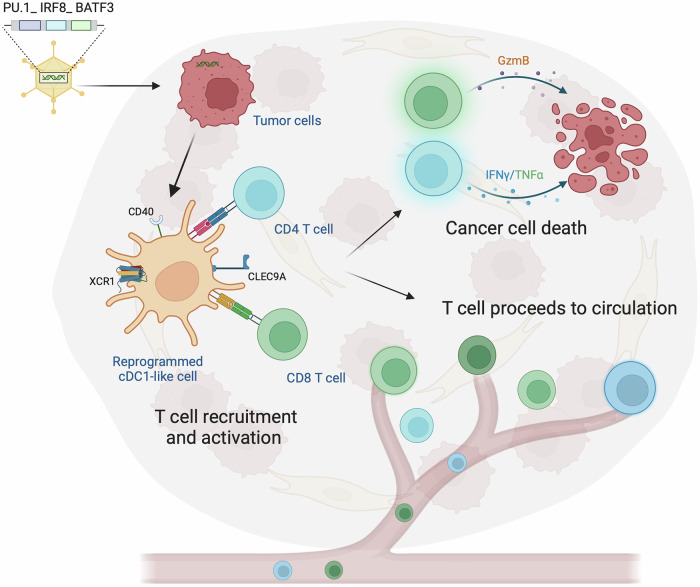
Immune activation by reprogrammed cDC1-like cells. Transcription factors PU.1, IRF8, and BATF3, delivered into tumor cells via viral vectors, reprogram these cells into type 1 conventional dendritic cell (cDC1)-like antigen-presenting cells. These reprogrammed cells are characterized by the expression of XCR1, CLEC9A, MHC class I/II, and CD40. The reprogrammed cDC1-like cells recruit and activate CD4^+^ and CD8^+^ T cells, inducing robust antitumor immune responses. T cells mediate tumor cell killing via the release of Granzyme B, as well as the secretion of IFN-γ and TNF-α. Effector and memory T cells are mobilized into circulation, contributing to systemic antitumor immunity and promoting an abscopal effect, suggesting the potential for broad tumor control. Created with BioRender.com

By reprogramming tumor cells to actively present antigens and recruit immune cells in vivo, Ascic and colleagues offer an innovative solution to one of the major limitations of current immunotherapy approaches: the lack of functional APCs within many tumors. Building up on the concept that was already introduced by Spranger et al. in 2017,^5^ this in situ reprogramming strategy could theoretically boost all types of immunotherapies. Their data suggest that, in general, patients receiving immune checkpoint blockade (ICB) could benefit from the treatment, and it may also help improve outcomes for patients who respond poorly to ICB. Since this therapy is entirely new, more data is required to assess its potential applications. This includes investigating the methods of vector administration, possible side effects, toxicity, and its potential use in combination with other immunotherapies, like CAR T cell therapy, to improve efficacy against solid tumors. Furthermore, additional research into the mechanisms of tumor cell-mediated antigen cross-presentation in vivo would be highly beneficial. Specifically, more studies are needed to gain a deeper understanding of how immune evasion mechanisms within the TME influence or impair the antigen presentation abilities of reprogrammed cells.

Nevertheless, turning cancer cells into APCs within the TME offers a potentially more accessible, “off-the-shelf” solution that could bypass the logistical challenges of ex vivo cell manufacturing. Ascic et al. provide evidence that this approach can make the tumor itself an active participant in its immune attack by transforming cancer cells into immune activators. This breakthrough could open new therapeutic avenues in which tumors are reprogrammed into allies in the fight against cancer, offering a promising new strategy for more effective and accessible cancer immunotherapies.

## References

[1] Ascic, E. et al. In vivo dendritic cell reprogramming for cancer immunotherapy. *Science* **386**, eadn9083 (2024). 39236156 10.1126/science.adn9083 PMC7616765

[2] Wculek, S. K. et al. Dendritic cells in cancer immunology and immunotherapy. *Nat. Rev. Immunol.* **20**, 7–24 (2020). 31467405 10.1038/s41577-019-0210-z

[3] Roberts, E. W. et al. Critical role for CD103(+)/CD141(+) dendritic cells bearing CCR7 for tumor antigen trafficking and priming of T cell immunity in melanoma. *Cancer Cell* **30**, 324–336 (2016). 27424807 10.1016/j.ccell.2016.06.003 PMC5374862

[4] Salmon, H. et al. Expansion and activation of CD103(+) dendritic cell progenitors at the tumor site enhances tumor responses to therapeutic PD-L1 and BRAF inhibition. *Immunity* **44**, 924–938 (2016). 27096321 10.1016/j.immuni.2016.03.012 PMC4980762

[5] Spranger, S., Dai, D., Horton, B. & Gajewski, T. F. Tumor-residing Batf3 dendritic cells are required for effector T cell trafficking and adoptive T cell therapy. *Cancer Cell* **31**, 711.e4–723.e4 (2017). 28486109 10.1016/j.ccell.2017.04.003 PMC5650691

